# Outcomes of ureteroscopy miniaturization on tissue damage and tissue hypoxia in a pig model

**DOI:** 10.1038/s41598-017-18885-8

**Published:** 2018-01-11

**Authors:** Juan P. Caballero-Romeu, Juan A. Galán-Llopis, Federico Soria, Esther Morcillo-Martín, Pablo Caballero-Pérez, Julia E. De La Cruz-Conty, Jesús Romero-Maroto

**Affiliations:** 1 Urology Department, Alicante University General Hospital, Alicante Institute for Health and Biomedical Research (ISABIAL-FISABIO Foundation), Alicante, Spain; 2 Urology Department, Vinalopó University Hospital, Elche, Alicante Spain; 3 Jesús Usón Minimally Invasive Surgery Center, Endoscopy Unit, Cáceres, Spain; 4 Community Nursing, Preventive Medicine and Public Health and History of Science Department, University of Alicante, Alicante, Spain; 5 Urology Department, San Juan University Clinic Hospital, Alicante, Spain. Miguel Hernández University, Alicante, Spain

## Abstract

Miniaturization of ureteroscopy materials is intended to decrease tissue damage. However, tissue hypoxia and the gross and microscopic effects on tissue have not been adequately assessed. We compared the gross and microscopic effects of micro-ureteroscopy (m-URS) and conventional ureteroscopy (URS) on the urinary tract. We employed 14 pigs of the Large White race. URS was performed in one of the ureters with an 8/9.8 F ureteroscope, while a 4.85 F m-URS sheath was used in the contralateral ureter. Gross assessment of ureteral wall damage and ureteral orifice damage was performed. For microscopic assessment hematoxylin-eosin staining and immunohistochemistry for detection of tissue hypoxia were conducted. Regarding the macroscopic assessment of ureteral damage, substantial and significant differences were recorded using URS (C = 0.8), but not with m-URS. Microscopic assessment after staining with hematoxylin-eosin revealed greater epithelial desquamation in the URS group (p < 0.05). Pimonidazole staining revealed greater hypoxia in the epithelial cells than in the remainder of the ureteral layers. We conclude that m-URS causes less damage to the ureteral orifice than URS. Histopathological findings show m-URS reduces ureteral epithelial damage compared with conventional ureteroscopy. Both URS and m-URS cause cellular hypoxia.

## Introduction

As a result of technological advances in ureteroscopes, imaging systems and ancillary devices, ureteroscopy has become one of the fundamental techniques used in endourology^1^. The new ureteroscopes allow us to maintain good operating conditions, with good irrigation flow, good visibility and the capacity to introduce ancillary instruments, using instruments of increasingly smaller calibers. Miniaturization should preserve the efficacy of the technique while reducing the complications derived from tissue damage caused by the ureteroscope within the urinary tract.

Damage to the ureteral wall has been associated with complications such as hematuria, postoperative edema or renoureteral colic. Vesicoureteral reflux, in turn, has been associated with loss of the valve mechanism of the orifice and intramural ureter. Furthermore, some authors have related the degree of ureteral damage to the need for postoperative ureteral stent placement and its duration^2^. On the other hand, ureteral stricture may also have an inflammatory and/or ischemic component^3^.

To further reduce ureteroscope caliber, micro-ureteroscopy (m-URS) was first described in 2014 as a ureteroscope using a 4.85 F (French) sheath of the micropercutaneous surgery set for the treatment of distal ureteral lithiasis in women, with satisfactory results^4,5^. Recently, Utanğaç *et al*. performed m-URS in a series of 11 children with a mean age of 55.1 months^6^. The authors reported that ureteral access in the pediatric patients could be carried out in all cases without preoperative ureteral stenting or dilatation of the ureteral orifice.

No comparative studies reporting scientific evidence at the micro- or macroscopic level of the ureteral effects of ureteroscopes of different calibers have been published. Therefore, the purpose of this experimental study was to determine whether micro-ureteroscopy performed with the 4.85 F access sheath of the micro-Perc® set (Polydiagnost, Germany) produces fewer adverse effects compared with a conventional 8/9.8 F ureteroscope, defined as less macroscopic damage to the ureteral wall, less macroscopic damage to the ureteral orifice (UO), less microscopic damage to the ureteral wall, and less tissue ischemia.

## Results

### Macroscopic assessment

**Figure 1:**
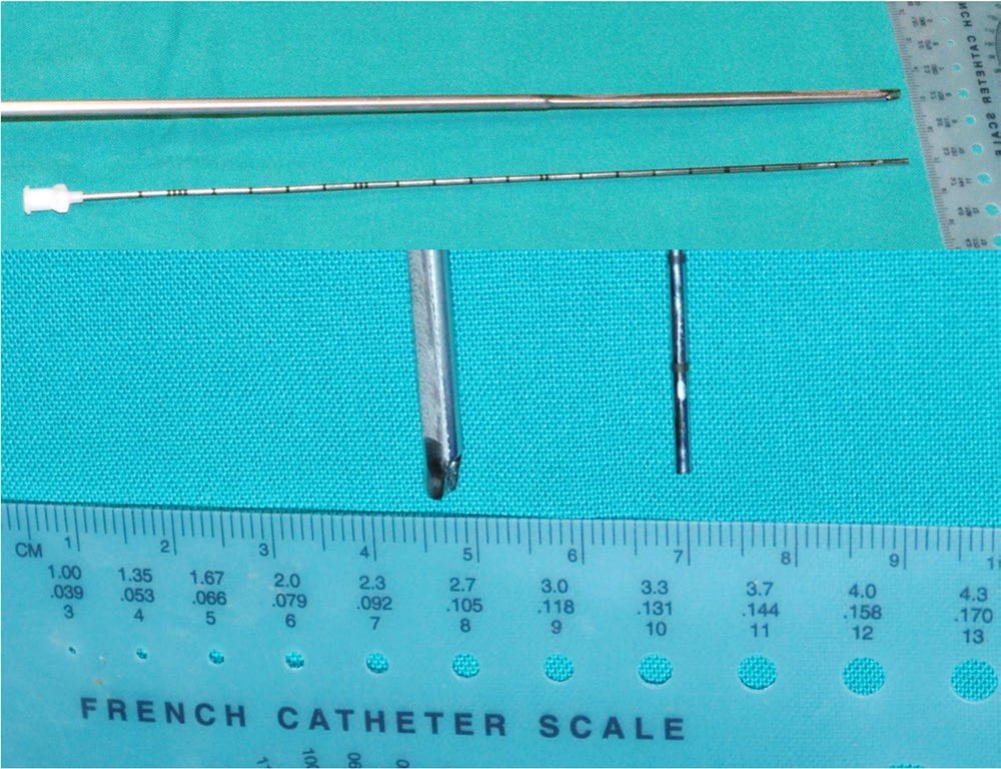
A longitudinal view of the m-URS ureteroscope and 4.85 F sheath is shown at the top. The tips of both instruments are shown at the bottom.

**Figure 2:**
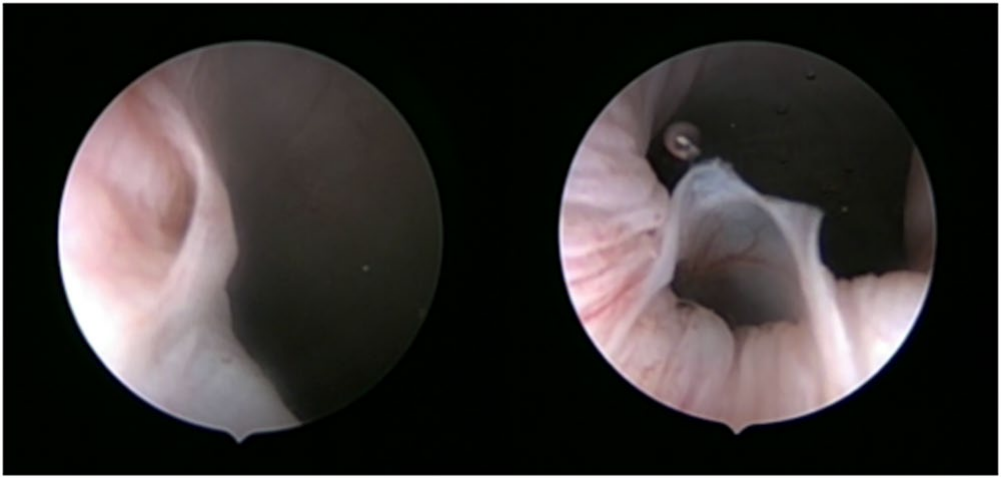
Comparison of the condition of the ureteral orifices after ureteral exploration. The ureteral orifice of a ureter explored with m-URS is shown at left. The ureteral orifice of a ureter explored with URS is shown at right.

In the blinded macroscopic assessment of ureteral damage, 84.62% of the ureteral orifices in which m-URS (Fig. 1) was performed showed no changes at the end of the procedure versus 15.38% in the URS group (p < 0.01) (Table 1). No differences between the baseline and final conditions of the ureteral orifice (UO) were observed with m-URS (C = 0.2). In contrast, substantial and significant differences were recorded using URS (p < 0.01), with an effect size of 0.8 (95% confidence interval [CI] 0.5-1) (Fig. 2). After m-URS, we identified 2 lesions of grade 1, while after URS, 9 lesions of grade 1 and 2 lesions of grade 2 were registered. The lesions, as scored by the Post-Ureteroscopic Lesion Scale (PULS), were greater in the URS group, with one grade 3 lesion (7.7%) versus one grade 1 lesion in the m-URS group (7.7%), though the differences between the two groups failed to reach statistical significance.

**Table 1:** Macroscopic evaluation of tissue damage.

			Scale n (%)				L		Effect size	
			0	1	2	3		Wil.+	C	95% CI
**Ureteral Orifice**										
Score	Baseline	URS	13 (100)	0	0	0	1	66.0**	0.8	(0.5; 1.0)
		m-URS	13 (100)	0	0	0	2	3.0	0.2	(0.0; 0.3)
	End	URS	2 (15.4)	9 (69.2)	2 (15.4)	0	5	0.0**	−0.7	(−0.9; −0.4)
		m-URS	11 (84.6)	2 (15.4)			6	0.0**	−0.7	(−0.9; −0.4)
**Ureter integrity**										
PULS	Baseline	URS	13 (100)	0	0	0	1	3.0	0.2	(0.0; 0.3)
		m-URS	13 (100)	0	0	0	2	1.0	0.1	(−0.1; 0.2)
	30 min	URS	12 (92.3)	0	0	1 (7.7)	3	1.0	0.1	(−0.1; 0.2)
		m-URS	12 (92.3)	1 (7.7)	0	0	4	1.0	0.1	(−0.1; 0.2)
	End	URS	12 (92.3)	1 (7.7)	0	1 (7.7)	5	1.5	0.1	(−0.2; 0.3)
		m-URS	12 (92.3)	1 (7.7)	0	0	6	1.5	0.1	(−0.2; 0.3)

Wil.+: Wilcoxon positive range statistic. C. Cliff’s delta. 95% CI: 95% confidence interval for Cliff’s delta. L: Legend, comparison between 1; Final URS vs Baseline URS,

2; Final m-URS vs Baseline mURS,3; 30 minutes URS vs Baseline URS,4; 30 minutes m-URS vs Baseline m-URS,

5; Final URS vs Final m-URS, 6; Final URS-Baseline URS vs Final m-URS – Baseline m-URS *p < 0.05, **p < 0.01.

### Microscopic ureteral damage assessment

**Figure 3:**
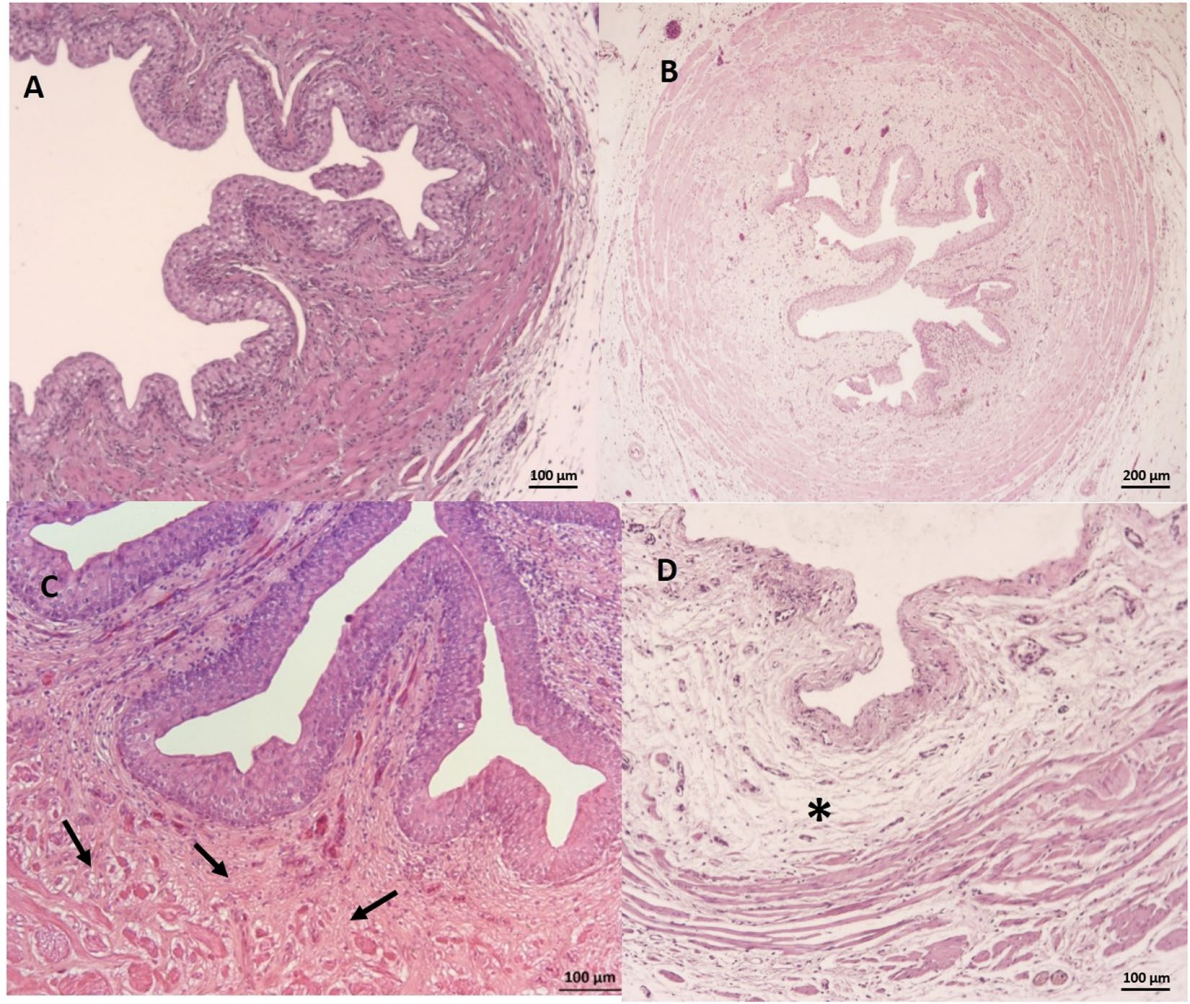
Ureter. (**A**) Intact epithelium (Lesion grade 0). (**B**) Loss of epithelium affecting less than half of the mucosa; focal loss (Lesion grade 1). (**C**) Intact epithelium of the ureter; lamina propria showing fibrosis extending towards the muscle layer (arrows). (**D**) In total, denuded mucosa; total loss of epithelium (Lesion grade 3). *Indicates moderate connective tissue edema of the lamina propria.

**Figure 4:**
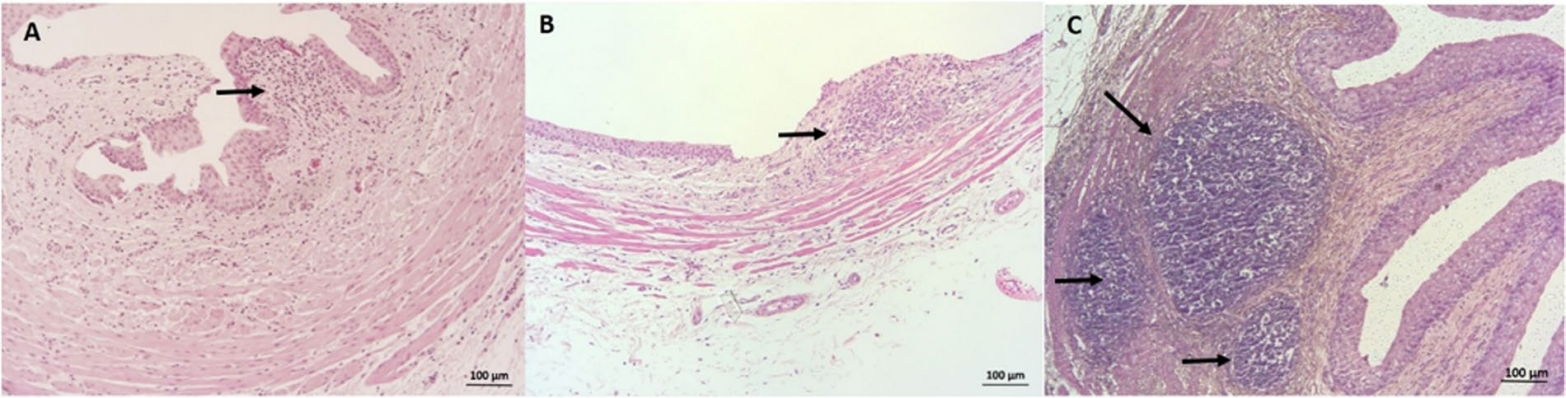
Ureter. (**A**) Loss of epithelium affecting less than half of the mucosa; focal loss (Lesion grade 1). The arrow indicates diffuse inflammatory infiltrate (**B** and **C**) Focal inflammatory infiltrates in the lamina propria.

Staining with hematoxylin-eosin (Table 2) revealed diffuse epithelial desquamation in 35.7% of the ureters in the URS group versus in 14.3% of the ureters in the m-URS group (p < 0.05). The effect of URS was substantial and significant, with C = 0.8 (0.5–0.9), while m-URS produced little and nonsignificant desquamation (Fig. 3). Ureteral edema, infiltration, fibrosis and congestion were identified in both groups in the proximal and distal ureter (Fig. 4). The differences in these measures between the two groups were not statistically significant. Ureteral bleeding was only identified focally in the URS group in two cases (14.3%). The presence of fibrosis in the muscle layer was focal in the proximal and distal ureter in both groups.

**Table 2:** Histopathological evaluation (hematoxylin-eosin staining).

		Absent	Focal	Diffuse	Severe	L	Wil.+	Effect size	
		n (%)	n (%)	n (%)	n (%)			C	95% CI
**EPITHELIUM**									
**Ureteral desquamation**									
Prox. u.	URS	3 (21.4)	11 (78.6)	0	0	1	55**	0.8	(0.5; 0.9)
	m-URS	3 (21.4)	11 (78.6)	0	0	2	24.5	0.3	(0.0; 0.6)
Distal u.	URS	0	4 (28.6)	5 (35.7)	5 (35.7)	3	0**	−0.5	(−0.8; −0.1)
	m-URS	1 (7.1)	10 (71.4)	2 (14.3)	1 (7.1)	4	0**	−0.5	(−0.8; −0.1)
**LAMINA PROPRIA**									
**Ureteral edema**									
Prox. u.	URS	4 (28.6)	8 (57.1)	2 (14.3)	0	1	45.5	0.2	(−0.2; 0.6)
	m-URS	3 (21.4)	8 (57.1)	3 (21.4)	0	2	27.5	0.0	(−0.4; 0.4)
Distal u.	URS	4 (28.6)	4 (28.6)	5 (35.7)	1 (7.1)	3	16.5	−0.1	(−0.5; 0.3)
	m-URS	3 (21.4)	8 (57.1)	3 (21.4)	0	4	16.5	−0.2	(−0.6; 0.2)
**Ureteral infiltration**									
Prox. u.	URS	5 (35.7)	6 (42.9)	3 (21.4)		1	7	−0.1	(−0.5; 0.3)
	m-URS	7 (50.0)	6 (42.9)	1 (7.1)		2	7	−0.1	(−0.5; 0.3)
Distal u.	URS	4 (28.6)	10 (71.4)	0		3	3.5	−0.3	(−0.6; 0.1)
	m-URS	8 (57.1)	6 (42.9)	0		4	3.5	0.0	(0.4; −0.4)
**Ureteral fibrosis**									
Prox. u.	URS	10 (71.4)	1 (7.1)	3 (21.4)		1	40.5	0.2	(−0.2; 0.6)
	m-URS	8 (57.1)	6 (42.9)	0		2	28	0.3	(−0.1; 0.6)
Distal u.	URS	5 (35.7)	8 (57.1)	1 (7.1)		3	30	0.0	(−0.3; 0.4)
	m-URS	5 (35.7)	7 (50.0)	2 (14.3)		4	30	0.0	(0.4; −0.4)
**Ureteral congestion**									
Prox. u.	URS	11 (78.6)	3 (21.4)	0		1	6	0.2	(−0.2; 0.5)
	m-URS	12 (85.7)	2 (14.3)	0		2	1.5	0.0	(−0.3; 0.3)
Distal u.	URS	9 (64.3)	4 (28.6)	1 (7.1)		3	3.5	−0.2	(−0.5; 0.1)
	m-URS	12 (85.7)	2 (14.3)	0		4	3.5	−0.2	(−0.5; 0.1)
**Ureteral bleeding**									
Prox. u.	URS	14 (100)	0			1	3	0.1	(−0.1; 0.3)
	m-URS	14 (100)	0			2	0	0.0	(0.0; 0.0)
Distal u.	URS	12 (85.7)	2(14.3)			3	0	−0.1	(−0.3; 0.1)
	m-URS	14 (100)	0			4	0	−0.1	(−0.3; 0.1)
**MUSCLE LAYER**									
**Fibrosis**									
Prox. u.	URS	12 (85.7)	2 (14.3)			1	7.5	0.1	(−0.2; 0.4)
	m-URS	11 (78.6)	3 (21.4)			2	9	0.1	(−0.3; 0.4)
Distal u.	URS	10 (71.4)	4 (28.6)			3	10.5	0.0	(−0.3; 0.3)
	m-URS	10 (71.4)	4 (28.6)			4	10.5	−0.1	(−0.4; 0.3)

Wil.+: Wilcoxon positive range statistic. C. Cliff’s delta. 95% CI: 95% confidence interval for Cliff’s delta. L: Legend, comparison between 1; Distal u. URS vs Prox. u. URS, 2; Distal u. m-URS vs Prox. u. m-URS, 3; Distal u. URS vs Distal u. m-URS,

4; Distal u. URS - Prox. u. Baseline URS vs Distal u. m-URS – Prox. u. m-URS, *p < 0.05, **p < 0.01.

Comparison of the number of cases of damage to the lamina propria caused by URS and m-URS (Table 3) showed no significant changes in damage with either method, though admittedly four or more types of different lesions were identified only after URS in 4 cases (28.5%).

**Table 3:** Evaluation of lamina propria damage according to procedure.

		Number of cases of damage						L	Wil.+	Effect size	
		0	1	2	3	4	5				
		n (%)	n (%)	n (%)	n (%)	n (%)	n (%)			C	95% CI
Prox. u.	URS	0	6 (42.9)	4 (28.6)	4 (28.6)	0	0	1	38.0	0.3	(−0.1; 0.7)
	m-URS	1 (7.1)	5 (35.7)	3 (21.4)	5 (35.7)	0	0	2	32.5	0.1	(−0.3; 0.5)
Distal u.	URS	0	2 (14.3)	7 (50.0)	1 (7.1)	3 (21.4)	1 (7.1)	3	22.5	0.1	(−0.3; 0.5)
	m-URS	1 (7.1)	3 (21.4)	5 (35.7)	5 (35.7)	0	0	4	37.0	−0.2	(−0.6; 0.2)

Wil.+: Wilcoxon positive range statistic. C. Cliff’s delta. 95% CI: 95% confidence interval for Cliff’s delta. L: Legend, comparison between 1; Distal ureter URS vs Proximal ureter URS, 2; Distal ureter m-URS vs Proximal ureter m-URS, 3; Distal ureter URS vs Distal ureter m-URS,

4; Distal ureter URS - Proximal ureter. Baseline URS vs Distal ureter m-URS – Distal ureter. m-URS, *p < 0.05, **p < 0.01.

### Hypoxia assessment

**Figure 5:**
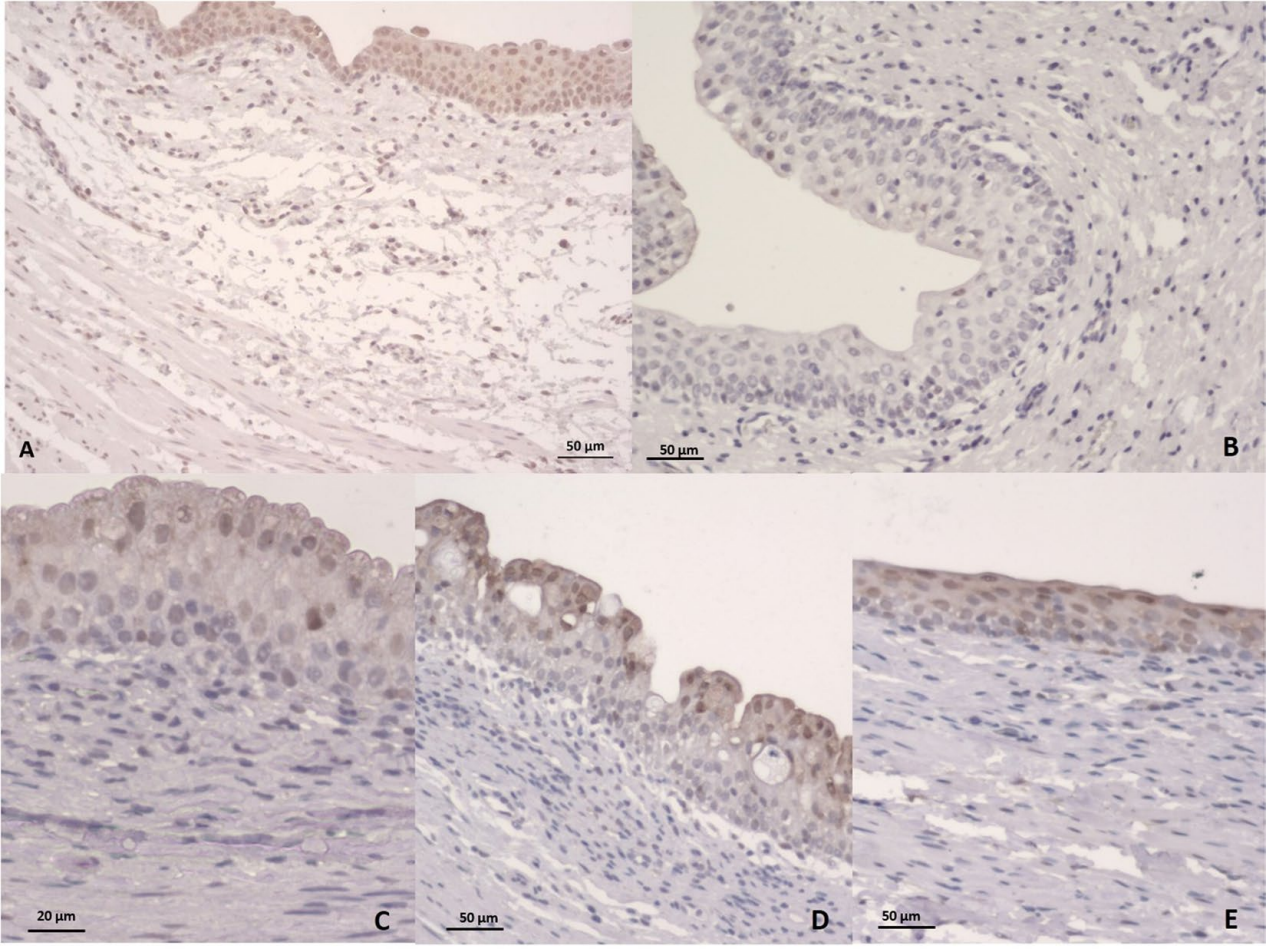
Ureter. (**A**) Positive pimonidazole immunohistochemical staining at the epithelial level (nucleus and cytoplasm), in cells of the lamina propria and nuclei of muscle cells. (**B**) Negative pimonidazole immunohistochemical staining. (**C**) Pimonidazole immunohistochemical staining at the epithelial level (positive nucleus and negative cytoplasm). (**D**) Positive pimonidazole immunohistochemical staining at the epithelial level (nucleus and cytoplasm), negative in the lamina propria. (**E**) Positive pimonidazole immunohistochemical staining at the epithelial level (nucleus and cytoplasm), negative in the lamina propria. See flat epithelium (dilated ureter).

**Figure 6:**
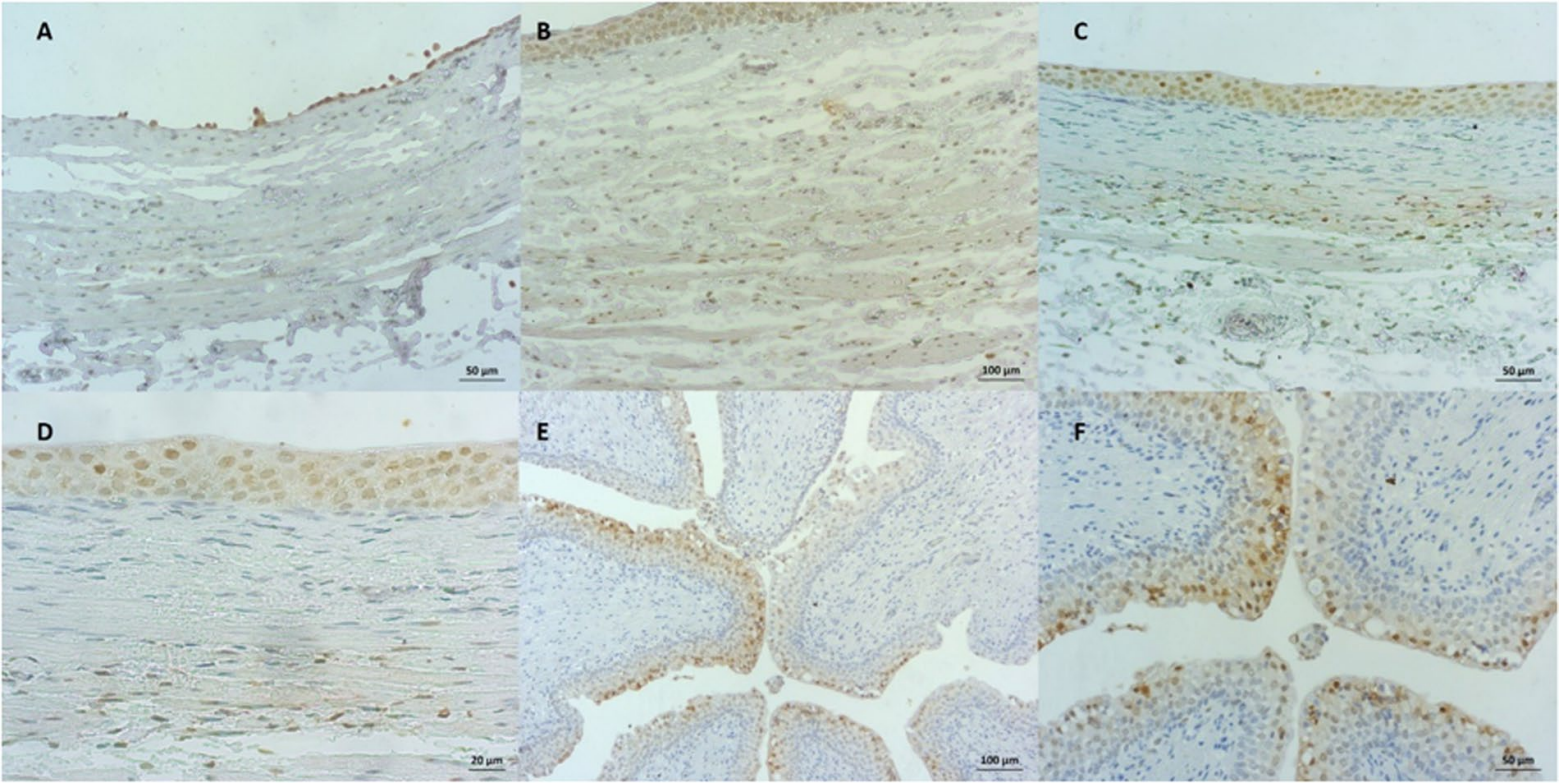
Ureter. (**A**) Positive pimonidazole immunohistochemical staining of the almost entirely lost epithelium (nucleus and cytoplasm) and in some cells of the lamina propria. (**B**) Positive pimonidazole immunohistochemical staining at the epithelial level (nucleus and cytoplasm), in cells of the lamina propria and nuclei of muscle cells. (**C**) Positive pimonidazole immunohistochemical staining at the epithelial level (nucleus and cytoplasm of some cells) and in nuclei of muscle cells. (**D**) Amplification of image C. (**E**) Positive pimonidazole immunohistochemical staining of some epithelial cells, negative in the lamina propria. Note the difference between positive and negative cells. (**F**) Amplification of image (**E**) Positive and negative cells in the same sample. Positive nucleus and positive and negative cytoplasm.

Pimonidazole staining (Table 4) revealed greater hypoxia in the nucleus and cytoplasm of the epithelial cells than in the remainder of the ureteral layers (Fig. 5). However, epithelial alteration in the proximal and distal ureteral zones was very similar with both URS and m-URS. Hypoxia affected 85.7% of nuclei in both groups in the proximal ureter. In the distal ureter, hypoxia was absent in 18.2% of cases in the URS group and 7.1% of cases in the m-URS group (NS) (Fig. 5). Pimonidazole staining was present in the cytoplasm in 64.3% of cases in the proximal ureter of the URS group and 57.2% in the m-URS group (NS). In the distal ureter, hypoxia was absent in 54.4% of cases in the URS group and in 28.6% in the m-URS group. The two techniques likewise showed no statistically significant differences in the lamina propria or the muscle or serosal layer between the proximal and distal ureteral segments (Fig. 6).

**Table 4:** Immunohistochemical evaluation.

		Absent	Present in few	Present in most	L	Wil.+	Effect size	95% CI
		n (%)	n (%)	n (%)			C	
**EPITHELIUM**								
**Nucleus**								
Prox. u.	URS	2 (14.3)	5 (35.7)	7 (50.0)	1	0	−0.1	(−0.5; 0.3)
	m-URS	2 (14.3)	6 (42.9)	6 (42.9)	2	25	0.0	(−0.4; 0.4)
Distal u.	URS	2 (18.2)	5 (45.5)	4 (36.4)	3	3	0.1	(−0.3; 0.5)
	m-URS	1 (7.1)	7 (50.0)	6 (42.9)	4	6	0.2	(−0.2; 0.6)
**Cytoplasm**								
Prox. u.	URS	5 (35.7)	3 (21.4)	6 (42.9)	1	0*	−0.2	(−0.5; 0.2)
	m-URS	6 (42.9)	4 (28.6)	4 (28.6)	2	12	0.1	(−0.4; 0.5)
Distal u.	URS	6 (54.5)	2 (18.2)	3 (27.3)	3	10*	0.2	(−0.2; 0.5)
	m-URS	4 (28.6)	7 (50.0)	3 (21.4)	4	10*	0.4	(0.0; 0.7)
**LAMINA PROPRIA**								
**Cells**								
Prox. u.	URS	9 (64.3)	5 (35.7)	0	1	12	0.2	(−0.2; 0.5)
	m-URS	7 (50.0)	7 (50.0)	0	2	18	0.0	(−0.4; 0.3)
Distal u.	URS	7 (50.0)	6 (42.9)	1 (7.1)	3	6	−0.1	(−0.4; 0.3)
	m-URS	8 (57.1)	5 (35.7)	1 (7.1)	4	3	−0.2	(−0.5; 0.3)
Substance								
Prox. u.	URS	14 (100)	0	0	1			
	m-URS	14 (100)	0	0	2			
Distal u.	URS	14 (100)	0	0	3			
	m-URS	14 (100)	0	0	4			
**MUSCLE LAYER**								
**Nucleus**								
Prox. u.	URS	7 (50.0)	7 (50.0)	0	1	4	0.0	(−0.4; 0.4)
	m-URS	12 (85.7)	2 (18.2)	0	2	10	0.2	(−0.1; 0.5)
Distal u.	URS	8 (57.1)	4 (28.6)	2 (18.2)	3	5	0.0	(−0.4; 0.4)
	m-URS	9 (64.3)	2 (18.2)	3 (21.4)	4	2	0.2	(−0.1; 0.5)
Cytoplasm								
Prox. u.	URS	14 (100)	0	0	1			
	m-URS	14 (100)	0	0	2			
Distal u.	URS	14 (100)	0	0	3			
	m-URS	14 (100)	0	0	4			
**SEROSA**								
**Serosa**								
Prox. u.	URS	10 (71.4)	2 (18.2)	2 (18.2)	1	7.5	0.0	(−0.3; 0.3)
	m-URS	11 (78.6)	2 (18.2)	1 (7.1)	2	5	0.0	(−0.3; 0.3)
Distal u.	URS	10 (71.4)	2 (18.2)	2 (18.2)	3	2.5	−0.1	(−0.4; 0.3)
	m-URS	11 (78.6)	2 (18.2)	1 (7.1)	4	7.5	−0.1	(−0.4; 0.3)

Wil.+: Wilcoxon positive range statistic. C. Cliff’s delta. 95% CI: 95% confidence interval for Cliff’s delta. L: Legend, comparison between 1; Distal u. URS vs Prox. u. URS, 2; Distal u. m-URS vs Prox. u. m-URS, 3; Distal u. URS vs Distal u. m-URS,

4; Distal u. URS - Prox. u. Baseline URS vs Distal u. m-URS – Prox. u. m-URS, *p < 0.05, **p < 0.01.

## Discussion

Ureteroscopy is a minimally invasive surgical technique. Although routine postoperative stenting is no longer recommended, a multicenter study demonstrated that, after 4475 ureteroscopies performed to treat distal ureteral stones, a double J stent was placed in 54.7% of patients^7^. Double J stents significantly reduce the quality of life of patients^8^. Micro-ureteroscopy was developed to reduce UO dilation and ureteral wall damage and, thus, to diminish postoperative stenting.

This study revealed that ureteral orifices in the m-URS group showed less damage. Damage to the UO is a key factor in the development of renoureteral colic episodes in the immediate postoperative period of ureteroscopy. The mucosal inflammatory reaction can significantly reduce the diameter of the distal ureter and cause flank pain^9–11^. Matani *et al*. found that 11.9% of 319 patients subjected to uncomplicated ureteroscopy required emergency placement of a stent less than 24 hours after the procedure^12^. The authors identified ureteral edema as one of the causes of the obstruction. The use of m-URS, therefore, could help avoid unnecessary ureteral stent placement in the absence of other risk factors for the development of post-ureteroscopy renoureteral colic episodes.

Furthermore, m-URS is the same caliber in its entire length unlike conventional ureteroscopes. Utanğaç *et al*. considered this finding to be relevant, even in comparison with 4.5/6.5 F ureteroscopes in pediatric patients, where the difference from m-URS was 25% at 5 cm from the tip of the instrument^5^. The decrease in instrument caliber from URS to m-URS was 39% at the tip of the instrument and 51% in the more proximal portion. m-URS might avoid the UO occlusion, and hence reduce renal intrapelvic pressure levels.

According to the authors of the PULS, grades 1 and 2 should be regarded as inherent to ureteroscopy and may imply the placement of a ureteral stent for some days, without being able to specify the exact duration of stenting^2,13^. The present study shows that m-URS offers advantages over URS in reducing ureteral epithelial desquamation. However, this difference was not noted by the participating surgeons. Epithelial desquamation as evidenced by the histopathology might not be assessable with the PULS or might not be clinically significant. In any case, the lesser epithelial damage observed in the hematoxylin-eosin staining could imply a decrease in the use of postoperative ureteral stents - with the consequent reduction of morbidity, improvement of patient quality of life^7^ and lowered costs associated with the endourological treatment of lithiasis.

Regarding the hypoxia assessment, one of the postulated causes of ureteral stricture is ischemia secondary to compression of the endoscopic material upon the ureteral wall. Lallas *et al*. used Doppler ultrasound to measure ureteral blood flow in a porcine model^14^. The authors compared three groups involving different caliber ureteral access sheaths (from 10–12 F to 14–16 F). No significant decrease in arterial flow could be demonstrated between the three groups. However, our study shows that oxygen supply at the cellular level is at least temporarily insufficient. A surprising finding was that a greater prevalence of epithelial cell nuclei with oxygen saturation levels <10 mmHg was noted in the proximal ureter not in contact with the ureteroscope than in the sections that came into contact with URS or m-URS. This only occurred at the epithelial level. In this regard, although transient and limited to the epithelium, ischemia caused by the intraluminal hydrostatic pressure might be more significant than the pressure exerted by the instruments. Further studies will be needed to determine the roles of other factors related to tissue hypoxia.

Some limitations of the study are to be acknowledged: firstly, the statistical power of the study might be insufficient to detect certain differences between the two techniques. On the other hand, we feel that the PULS may be useful in deciding whether to place a postoperative ureteral stent. However, this scale does not distinguish between lesions already present before treatment for lithiasis and lesions observed at the end of the procedure. Moreover, the study does not contemplate stone fragmentation, which reduces the likeliness of ureteral wall damage, or the damage caused by renal lithiasis itself. Lastly, our study does not allow us to determine the mechanism underlying cellular ischemia at the proximal ureter with either URS or m-URS.

In summary, our study shows that m-URS causes less damage to the ureteral orifice than conventional ureteroscopy. The damage caused at the ureteral level is not noticeable when assessed using the PULS. The histopathological findings show m-URS reduces ureteral epithelial damage compared with conventional ureteroscopy. Both URS and m-URS cause cellular hypoxia, though this phenomenon cannot be exclusively attributed to endoscope contact with the ureteral wall.

## Methods

A porcine model involving 14 healthy female pigs (Large White race) weighing 30–35 kg was used in this experimental study. Three phases were established, as shown in the flow diagram (Fig. 6). The Institutional Ethics Committee for Animal Research of Jesús Usón Minimally Invasive Surgery Center approved the experimental protocol. The principles of laboratory animal care (NIH publication no. 86–23, revised 1985) were followed, as was the current version of the European Union Laws on the protection of animals used for scientific purposes.

### Phase I: Animal model preparation

The animals were subjected to inhaled general anesthesia. To rule out abnormalities that could interfere with the results of the study, baseline evaluation was carried out in all animals. First, a blood sample was drawn for hematological and biochemical analyses. Second, ultrasonographic (US) evaluation of both kidneys was performed, and the Hydronephrosis Score (HS) was recorded according to the Society of Fetal Urology classification^15^. Following this, the mucosal alterations at the ureteral orifice were categorized according to a validated UOScore^16^ (UO_0_ = normal ureteral orifice; UO_1_ = enlarged ureteral orifice with light surrounding inflammatory reaction; UO_2_ = enlarged ureteral orifice with moderate surrounding inflammatory reaction; UO_3_ = enlarged ureteral orifice with severe surrounding inflammatory and cystic reaction). The urinary tract evaluation finished with a compression cystography to rule out vesicoureteral reflux (VUR), as previously described^16^.

Once the baseline evaluation was completed, a tissue ischemia marker, pimonidazole diluted in 100 ml of saline solution, was then injected as a slow intravenous infusion. Pimonidazole (Hypoxyprobe kit, NPI Inc. USA) was administered at a dose of 0.3–0.5 g/m^2^ body surface area via the intravenous route.

### Phase II: Ureteroscopy/micro-ureteroscopy

After Phase I of the study, we started Phase II, which consisted of the endoscopic exploration of both ureters in each pig. In 14 ureters (7 rights and 7 lefts ureters) the ureteroscopy was carried out using a conventional 8/9.8 F ureteroscope (Richard Wolf Medical Instruments^©^, Knittlingen, Germany), forming the URS group. The other 14 ureters were explored with micro-ureteroscopy (m-URS group) with the MicroPerc® set (Polydiagnost, Hallbermoos, Germany). Specifications of both ureteroscopes are shown in Fig. 1.

In the m-URS group, access to the ureteral orifice was carried out without a safety guide, while in the URS group access to the ureteral orifice was carried out using a 0.035” hydrophilic safety guide. Following insertion of the ureteroscope, the distance from the tip of the endoscope to the external urethral meatus of the animal was kept fixed at 21 cm. In all cases, the first step was the evaluation of the ureteral orifice (UO Score), and the endoscopic appearance of the ureteral lumen was scored by the PULS^13^. Small movements were made in the craniocaudal direction, and a straight-tipped 230–270 µm laser fiber was used to simulate a therapeutic intervention. The irrigation system was always achieved with a gravity irrigation system, placing a normal saline bag 70 cm above the level of the animal, and the total irrigation saline volume was recorded.

The exploration time was determined according to scientific literature, so URS group was 60 min and m-URS was 45 min^17^. Once the exploration was completed, ureteral wall injury was scored by PULS and the ureteral orifice damage by the UO Score.

The experimental phase was carried out by two surgeons with extensive experience in both endourological techniques. The two study techniques, ureteroscopy and micro-ureteroscopy, were alternated in each animal. Accordingly, each surgeon performed 7 URS and 7 m-URS, representing a total of 28 procedures in 14 animals.

The m-URS system consisted of a sheath measuring 22.5 cm in length and with a caliber of 4.85 F. The caliber was uniform over the entire length. The sheath, in turn, was fitted to a 3-Luer-Lock. The irrigation solution entered through one of the lateral arms of the adaptor. Optics measuring 0.9 mm in diameter with 120° vision and a resolution of 10,000 pixels was inserted through the central channel. A Tuohy Borst adaptor (Cook Medical, Bloomington, USA) was fitted to the third channel for the insertion of a laser fiber. The ureteral wall was evaluated at the start and end of the procedure.

### Phase III: *Ex-vivo* experiments

The animals were euthanized after completing both explorations. The experimental study was completed by removing the urinary tract en bloc. Microscopic evaluation was carried out by a pathologist blinded to the group to which the samples belonged. An evaluation was made of the ureteral wall in contact with the ureteroscope or micro-ureteroscope. This segment is henceforth referred to as the distal ureter. A segment of ureter located above the study zone and 1 cm below the ureteropelvic junction was used as a control. This section, which was not manipulated during the exploration, is henceforth referred to as the proximal ureter. The minimum distance between the ureteropelvic junction and the explored zone was approximately 9 cm. A pathological classification was used with the hematoxylin-eosin–stained specimens to score 6 parameters from 0–3 (where 0 = no changes and 3 = severe changes). Wall inflammation, fibrosis of the lamina propria, muscle fibrosis, integrity of the muscle layer and alterations of the serosal layer were studied. The immunohistochemical analysis, in turn, assessed the presence or absence of staining in each of the ureteral layers, considering the cell nucleus and cytoplasm separately. Staining was scored as absent, present in a few cells, or present in most cells.

### Statistical analysis

The study was designed based on conservative criteria conditioned to ethics in animal experimentation. A total of 14 individuals was calculated as the sample size needed for a global frequency of complications associated with the procedure of 25%^18^, an expected reduction of complications to 1%, a statistical power of 75% and an alpha error of 0.1.

In the present study, all the outcome variables were ordinal; Cohen’s delta, therefore, was the best choice for contrasting the effect size between the two procedures. However, this effect measure is based on averages and variances and therefore constitutes a parametric-effect indicator. Our sample size was small, and Cohen’s delta was, therefore, not the best option. Cliff’s delta (C) was best suited for our purposes. In all cases, Cliff’s delta lies between 1 and −1, where 1 and −1 are interpreted as representing extreme effect sizes, while 0 is indicative of a null effect. Values of about ±0.11 represent a small effect, ±0.28 is indicative of a medium effect, and ±0.43 is indicative of a substantial effect. Positive values of Cliff’s delta were indicative of increased damage when comparing the two procedures or comparing them with their controls. In contrast, negative values were indicative of lesser damage. Both Cliff’s delta and the corresponding confidence interval were calculated using the R statistical application and its “effsize” package (version of November 2016).

Likewise, a comparison of paired means would not be the best option for detecting associations between the procedures used and the damage caused. With a sample size of 14 individuals per group, we decided to substitute such comparison with the Wilcoxon signed rank test. Both the SPSS statistics Version 15 (IBM, Armonk, USA) and the R statistical application were used for the contrasting hypotheses.

Comparisons were made between the cases and controls within each procedure; the cases of one procedure versus the cases of the other; and finally, between the cases and controls of the two procedures. This approach allowed us to consider the damage caused from the starting point, which was not necessarily the same in all cases. In the absence of damage in the controls of both procedures, the latter two comparisons would be equivalent.

### Data availability statement

All data are available upon request.

## Electronic supplementary material

Supplementary info/figures
