# Supplementary material for: Outcomes of ureteroscopy miniaturization on tissue damage and tissue hypoxia in a pig model
Source: Sci Rep. 2018 Jan 11;8:431. doi: 10.1038/s41598-017-18885-8 (PMC5765032; doi:10.1038/s41598-017-18885-8)

Title of the manuscript: Outcomes of ureteroscopy miniaturization on tissue damage and tissue hypoxia in a pig model.

Corresponding Author: Juan P. Caballero-Romeu; MD.

Affiliation: Urology Department, Alicante University General Hospital, Alicante Institute for Health and Biomedical Research (ISABIAL – FISABIO Foundation), Alicante, Spain.

Postal address:

Pintor Baeza núm. 12

03010 Alicante, Spain

Telephone number: +34.687.90.40.95

Fax number: +34.965.91.37.53

e-mail address: juanpablocaballero@gmail.com

Author: Juan A. Galán-Llopis; PhD.

Affiliation: Urology Department, Vinalopó University Hospital, Elche (Alicante), Spain.

Postal address: Carrer Tonico Sansano Mora, 14, 03293, Elche (Alicante), Spain.

E-mail address: jagalanllopis@gmail.com

Author: Federico Soria; PhD.

Affiliation: Jesús Usón Minimally Invasive Surgery Center, Endoscopy Unit, Cáceres, Spain

Postal address: Carretera N-521, km. 41,8. 10071, Cáceres (Spain)

E-mail address: fsoria@ccmijesususon.com

Author: Esther Morcillo-Martín; PhD.

Affiliation: Jesús Usón Minimally Invasive Surgery Center, Endoscopy Unit, Cáceres, Spain

Postal address: Carretera N-521, km. 41,8. 10071, Cáceres (Spain)

E-mail address: emorcillo@ccmijesususon.com

Author: Pablo Caballero-Pérez; PhD.

Affiliation: Community Nursing, Preventive Medicine and Public Health and History of Science Department, University of Alicante, Alicante, Spain.

Postal address: Carretera San Vicente del Raspeig, s/n, 03690 San Vicente del Raspeig, Alicante, Spain

E-mail address: pablo.caballero@ua.es

Author: Julia E. De La Cruz-Conty

Affiliation: Jesús Usón Minimally Invasive Surgery Center, Endoscopy Unit, Cáceres, Spain

Postal address: Carretera N-521, km. 41,8. 10071, Cáceres (Spain)

E-mail address: jecruz@ccmijesususon.com

Author: Jesús Romero-Maroto; Prof.

Affiliation: Urology Department, San Juan University Clinic Hospital, Alicante, Spain. Miguel Hernández University, Alicante, Spain.

Postal address: Crta. Nacional 340, Km 87, 03550 Sant Joan d'Alacant, Alicante, Spain

E-mail address: jromeroma@coma.es

Study protocol diagram.

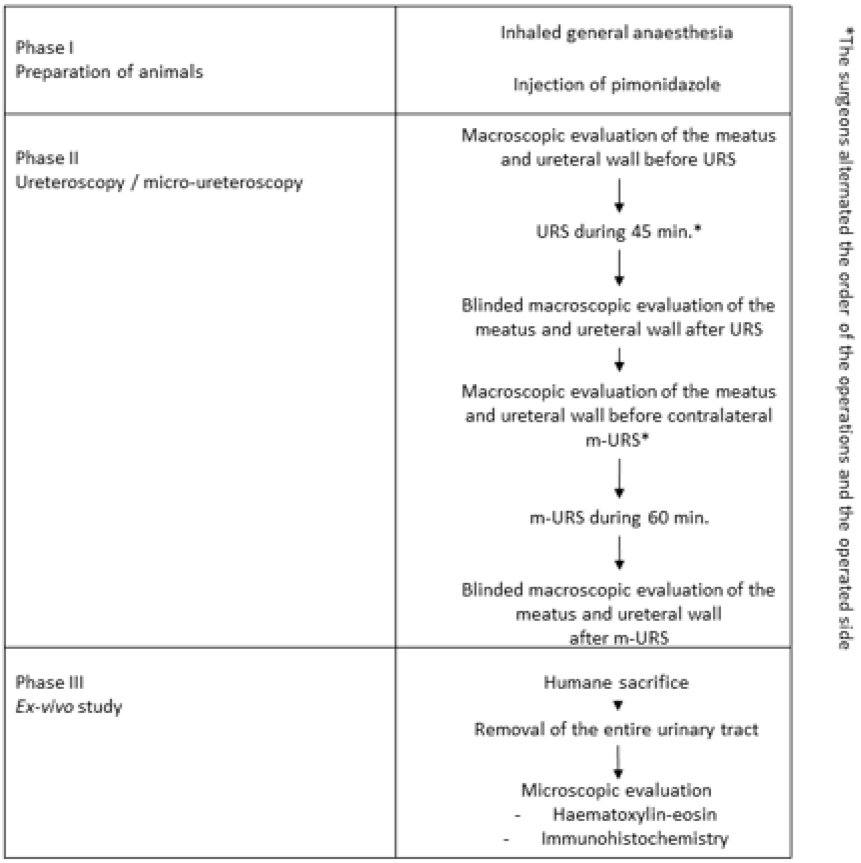

Micro-URS sheath inserted 21 cm in the sow. The proximal ureter does not come into contact with the sheath. White arrow: tip of the 4.85F sheath.

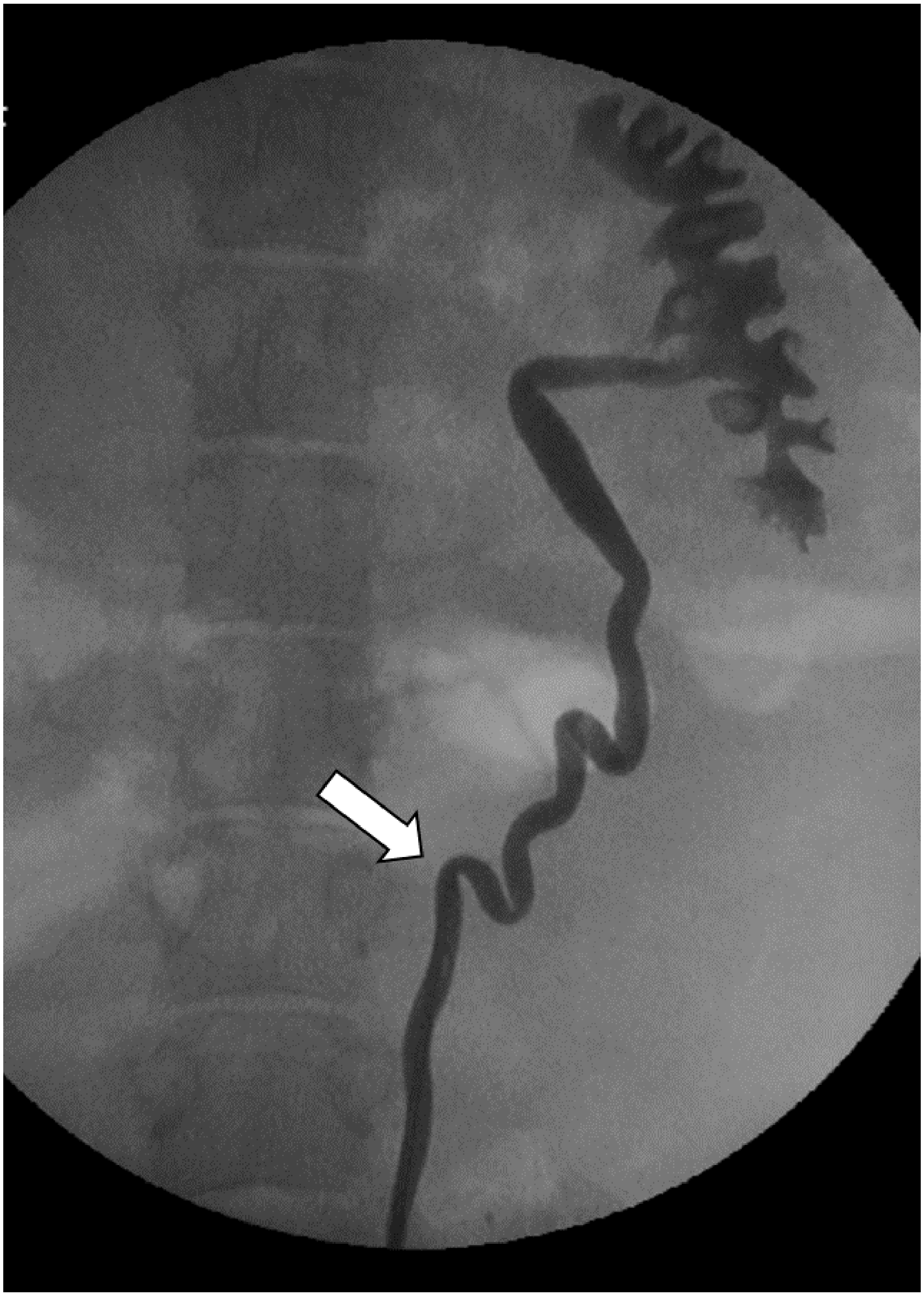

Supplement: Supplementary file 1 — Supplementary info/figures [file 41598_2017_18885_MOESM1_ESM.pdf]
